# Tolerogenic Dendritic Cells That Inhibit Autoimmune Arthritis Can Be Induced by a Combination of Carvacrol and Thermal Stress

**DOI:** 10.1371/journal.pone.0046336

**Published:** 2012-09-25

**Authors:** Rachel Spiering, Ruurd van der Zee, Josée Wagenaar, Dimos Kapetis, Francesca Zolezzi, Willem van Eden, Femke Broere

**Affiliations:** 1 Department of Infectious Diseases and Immunology, Utrecht University, Utrecht, The Netherlands; 2 Department of Biotechnology and Bioscience, Genopolis, University of Milano-Bicocca, Milan, Italy; University of Southern California, United States of America

## Abstract

Tolerogenic dendritic cells (DCs) can induce regulatory T cells and dampen pathogenic T cell responses. Therefore, they are possible therapeutic targets in autoimmune diseases. In this study we investigated whether mouse tolerogenic DCs are induced by the phytonutrient carvacrol, a molecule with known anti-inflammatory properties, in combination with a physiological stress. We show that treatment of DCs with carvacrol and thermal stress led to the mRNA expression of both pro- and anti-inflammatory mediators. Interestingly, treated DCs with this mixed gene expression profile had a reduced ability to activate pro-inflammatory T cells. Furthermore, these DCs increased the proportion of FoxP3^+^ regulatory T cells. *In vivo*, prophylactic injection of carvacrol-thermal stress treated DCs pulsed with the disease inducing antigen was able to suppress disease in a mouse model of arthritis. These findings suggest that treatment of mouse bone marrow derived DCs with carvacrol and thermal stress induce a functionally tolerogenic DC that can suppress autoimmune arthritis. Herewith carvacrol seems to offer novel opportunities for the development of a dietary based intervention in chronic inflammatory diseases.

## Introduction

Fully matured dendritic cells (DC) potently induce effector T cell responses via increased expression levels of co-stimulatory molecules and pro-inflammatory cytokines such as interleukin (IL)-12, IL-6 and tumor necrosis factor α (TNFα). In contrast, tolerogenic DCs are able to reduce pro-inflammatory T cell responses and induce regulatory T cells or T cell anergy. Several phenotypic characteristics have been described for tolerogenic DCs. For example low or intermediate expression levels of maturation markers, expression of IDO or secretion of IL-10 are all features of tolerogenic DCs [1]. Due to their ability to dampen immune responses by the induction of antigen specific regulatory T cells, tolerogenic DCs have gained interest as a therapeutic strategy in autoimmune diseases such as rheumatoid arthritis (RA) [2]–[7]

RA is a chronic autoimmune disease characterized by joint inflammation resulting in progressive cartilage and bone erosion. Inflammatory cytokines such as TNFα and IL-1β and increased levels of the Th1 cytokines are involved in its pathogenesis. Administration of biologics such as anti-TNFα, has proven to be an efficient therapy for RA, but this systemic suppression of the immune response has unwanted consequences for host defense mechanisms. In contrast, antigen specific therapies are expected to locally suppress immune responses and this approach could obviate these severe side effects.

Recently, we reported that intragastric administration of carvacrol, a component of the essential oil of *Oreganum* species, suppressed proteoglycan induced arthritis (PGIA) a chronic, progressive and self-maintaining T cell dependent, antibody-mediated murine model for RA. [8]. Carvacrol was shown to be a potent co-inducer of heat shock protein 70 (HSP70) in various antigen presenting cells. In mammalian cells HSP70 expression can be induced e.g. by thermal stress (TS) and TS has been described to inhibit as well as to induce the production of several pro-inflammatory cytokines depending on culture conditions and cell type [9]–[12]. Oral carvacrol treatment in mice induced a regulatory T cell response that could transfer protection in the PGIA model [8]. Moreover, carvacrol has been described to exhibit anti-bacterial properties [13], anti-oxidant activity [14] and anti-inflammatory qualities. Carvacrol was able to suppress lipopolysaccharide (LPS) induced cyclooxygenase (COX)-2 expression in human macrophages, possibly by the induction of the anti-inflammatory transcription factors peroxisome proliferator-activated receptor (PPAR)-α and PPAR-γ [15]. In addition, carvacrol also directly inhibited the production of prostaglandin E_2_ (PGE_2_) [16].

For several reasons, oral administration of carvacrol may not be the best approach. For the application for humans, the amount of carvacrol needed to be effective may be too high and because of its anti-bacterial properties high dosages of carvacrol could negatively affect the intestinal flora. Therefore, we explored the effect of carvacrol in the induction of a tolerogenic phenotype in DCs in the PGIA model. We found that both TS and carvacrol in combination with TS induced some of the phenotypic characteristics of a tolerogenic DC. However, functionally only carvacrol-TS treated DCs were able to suppress autoimmune arthritis in the PGIA model, possibly by the induction of high levels of stress related immuno-regulatory molecules.

## Materials and Methods

### Ethics statement

The animal studies and experiments were approved by the Utrecht University Animal Experimental Committee.

### Carvacrol/thermal stress treatment of BMDC

Bone marrow derived dendritic cells (BMDC) were isolated from the bone marrow of 10–15 week old BALB/c mice and cultured in IMDM (Gibco) supplemented with 10% FBS (Lonza), 100 units/ml penicillin, 100 µg/ml streptomycin and 5×10^−5^ M β-mercaptoethanol in the presence of 20 ng/ml GM-CSF (Cytogen) [17]. On day 8 BMDC were seeded in 6 wells plates at 1×10^6^ cells/ml. The next day, cells were treated with 0.1 mM carvacrol (Sigma) dissolved at 100 mM in ethanol. Control cultures were incubated with medium alone or 0.1% ethanol. After two hours at 37°C plates were sealed and placed in a 42.5°C preheated water bath for one hour (TS treatment). Cells were able to recover at 37°C for different time points. With this treatment protocol we have obtained optimal stimulation of Hsp70 in previous experiments [18].

### Mice

Female BALB/c mice were purchased from Charles River Laboratories. DO11.10 mice, transgenic for the pOVA (ovalbumin 323–339) specific T cell receptor (TCR), were bred and kept under standard conditions and received water and food *ad libitum*.

### RNA isolation for microarray

BMDC were treated as described above. Cells were harvested 0.5 hour after carvacrol addition or TS treatment. Total RNA was extracted from 2×10^6^ BMDC using the double extraction protocol: first by acid guanidinium thiocyanate-phenol-chloroform extraction (Trizol Invitrogen) followed by a Qiagen RNeasy clean-up procedure.

### Microarray expression profiling

Three independent (n = 3) replicates were performed for each experimental condition (controls, carvacrol, TS and carvacrol-TS). Total RNA was processed for the use onGeneChip® Mouse Genome 430A 2.0 Array (Affymetrix, Santa Clara, CA) containing 22.000 murine probe sets which interrogates approximately 14.000 transcripts. RNA quality controls, hybridization, washing, staining, and scanning procedures were performed by Genopolis (University of Milano-Bicocca). In brief, RNA purity was assessed by spectrophotometer (Nanodrop) and integrity by Agilent Bioanalyzer. 0.3 µg RNA for each sample were used in a reverse transcription reaction (MessageAmp III RNA Amplification kit, Ambion) to generate first-strand cDNA. After second-strand synthesis, double strand cDNA was used in an *in vitro* transcription reaction to generate biotinylated cRNA. After purification and fragmentation and relative quality control checks by Agilent Bioanalyzer, biotinylated cRNA was used for hybridization. Fragmented cRNAs were hybridized to the standard arrays for 16 hours at 45°C; the arrays were then washed and stained using the GeneChip Fluidics Station 450 and scanned using GeneChip Scanner 3000. The images were analyzed using Affymetrix GCOS software version 1.4.

### Analysis of array data

Data handling was done using Bioconductor software [19]. The Robust Multi-array Analysis [20] method was employed to calculate probe set intensity. After checks of sample data to verify the quality of replicates, hierarchical clustering based on Pearson correlation coefficients was performed. The identification of differentially expressed genes was addressed using a linear modelling approach (Limma) and empirical Bayes methods [21] together with false discovery rate correction of the *p*-value [22]. Differentially expressed genes with *p*-values of ≤0.05 were selected. Probe sets were annotated following Affymetrix annotation files. Primary data are available in the public domain through ArrayExpress at http://www.ebi.ac.uk/arrayexpress under the accession number E-MEXP-3489. For data analysis Database for Annotation, Visualisation and Integrated Discovery (DAVID) was used.

### In vivo effect of carvacrol treated BMDC on antigen specific T cells

CD4^+^ pOVA-specific T cells were isolated from spleens of DO11.10 mice and labeled with 5,6-carboxy-succinimidyl-fluoresceine-ester (CFSE) as described before [23]. Acceptor BALB/c mice received intravenously 1×10^7^ CD4^+^ CFSE-labeled cells in 100 µl PBS. The subsequent day BMDC were treated as above, allowed to recover for four hours and pulsed for another two hours with 20 µg/ml pOVA prior to intraperitoneally transfer of 1×10^6^ BMDC to acceptor mice. Three days later mice were sacrificed and spleen and mesenteric lymph nodes (mLN) were isolated.

### Flow cytometric analysis of surface markers, HSP70 and FoxP3

BMDC were treated as described above. After overnight recovery cells were stained with APC-anti-CD11c (HL3) plus one of the following antibodies: PE-anti-CD40 (3/23), FITC-anti-CD86 (GL1) or PE-anti-I-Ad/I-Ed (M5/114) (BD Biosciences). To analyze HSP70 expression, cells were stained with APC-anti-CD11c followed by intracellular HSP70 staining [8]. Single cell suspensions of spleen and mLN were stained with pacific-blue labeled anti-CD4 (RM4-5) and APC-anti-KJ1.26 (OVA-TCR) (BD Biosciences). Additionally, FoxP3 staining was carried out with a FoxP3 (FJK-16S; PE labeled) staining kit as instructed (eBioscience). Flow-cytometry was performed on a FACSCanto (BD Biosciences).

### Analysis of antigen-specific T cell responses

Single cell suspensions of spleen and mLN were cultured in complete medium in flat-bottom plates (Corning) at 2×10^5^ cells per well, in the presence/absence of pOVA (20 µg/ml). After 72 hours supernatants were collected for cytokine assays. Fluoresceinated microbeads coated with capture antibodies for simultaneous detection of IL-6 (MP5-20F3), IL-2 (JES6-1A12), TNFα (G281-2626) (BD Biosciences) and IFNγ (AN-18) (homemade) were added to 50 µl of culture supernatant. Cytokines were detected by biotinylated antibodies IL-6 (MP5-32C11), IL-2 (JES6-5H4), TNFα (MP6-XT3), IFNγ (XMG1.2) and PE-labeled streptavidin (BD Biosciences) and analyzed on a Luminex model 100 XYP (Luminex). In parallel cells were used for RT-PCR quantification of mRNA of IFNγ, IL-1β, IL-10 and TGFβ.

### Analysis of mRNA expression by quantitative real time PCR

BMDC were treated as described above. BMDC, mLN or spleen cells were harvested at indicated time points and stored in 350 µl RLT with 1% β-mercaptoethanol. Total mRNA extraction with the RNeasy kit (Qiagen), on column DNAse treatment (Qiagen), and transcription into cDNA using the iScript^TM^ cDNA Synthesis kit (Bio-Rad) were carried out according to the manufacturer's protocol. Quantitative RT-PCR (3 minutes at 95°C and 40 cycles of 10 seconds 95°C and 45 seconds at 59.5°C) was performed in a Bio-Rad MyiQ iCycler (Bio-Rad). Amplification was done in a total volume of 25 µl using IQ^TM^ SYBR Green® Supermix (Bio-Rad Laboratories B.V.) with 0.25 µM final concentrations of primers (table 1). For each sample mRNA expression was normalized to the detected Ct value of HPRT and, for BMDC expressed relative to the average of the control group.

**Table 1:** Primer sequences used for quantitative real time PCR.

	Forward primer 5′–3′	Reverse primer 5′–3′
**HPRT**	CTG GTG AAA AGG ACC TCT CG	TGA AGT ACT CAT TAT AGT CAA GGG CA
**CCL2**	TTA AAA ACC TGG ATC GGA ACC AA	GCA TTA GCT TCA GAT TTA CGG GT
**CCL7**	GCT GCT TTC AGC ATC CAA GTG	CCA GGG ACA CCG ACT ACT G
**CCL12**	ATT TCC ACA CTT CTA TGC CTC CT	ATC CAG TAT GGT CCT GAA GAT CA
**LIF**	GCT ATG TGC GCC TAA CAT GAC	CGC TCA GGT ATG CGA CCA T
**AREG**	GGG GAC TAC GAC TAC TCA GAG	TCT TGG GCT TAA TCA CCT GTT C
**IL-10**	TGC CAA GCC TTA TCG GA	ACC TGC TCC ACT GCC TTG CT
**MT1**	AAG AGT GAG TTG GGA CAC CTT	GA GAC AAT ACA ATG GCC TCC
**HSP1A1**	AAG AAC GCG CTC GAA TCC TA	GAG ATG ACC TCC TGG CAC TTG T
**IFNγ**	TCA AGT GGC ATA GAT GTG GAA GAA	TGG CTC TGC AGG ATT TTC ATG
**IL-1β**	CAA CCA ACA AGT GAT ATT CTC CAT G	GAT CCA CAC TCT CCA GCT GCA
**TGFβ**	GCC CTG TAT TCC GTC TCC TCC TTG	CGT AAC CGG CTG CTG ACC

### In vivo treatment and arthritis induction

Arthritis was induced in female mice, 16 to 26 weeks old, using a standard immunization protocol by intraperitoneal injection of human PG (250 µg) and 2 mg dimethyldioctadecylammonium bromide (DDA) (Sigma) emulsified in 200 µl PBS on days 0 and 21 [24]. One day prior to the second PG/DDA immunization BMDC treated as described above were able to recover for four hours and then pulsed with 250 µg/ml PG. Two hours later 1×10^6^ BMDC were resuspended in 200 µl PBS and intraperitoneally injected into acceptor mice. As a control 200 µl PBS only was used. Onset and severity of arthritis was determined using a visual scoring system based on swelling and redness of paws [24].

### Statistical analysis

Statistical analysis was carried out using Prism software (version 4.00, Graphpad software Inc.). Significance level was set at p≤0.05 and one-tailed Student's t test was applied.

## Results

### Gene expression profiling of thermal stress (TS) and carvacrol-thermal stress (CTS) treated BMDC

**Figure 1:**
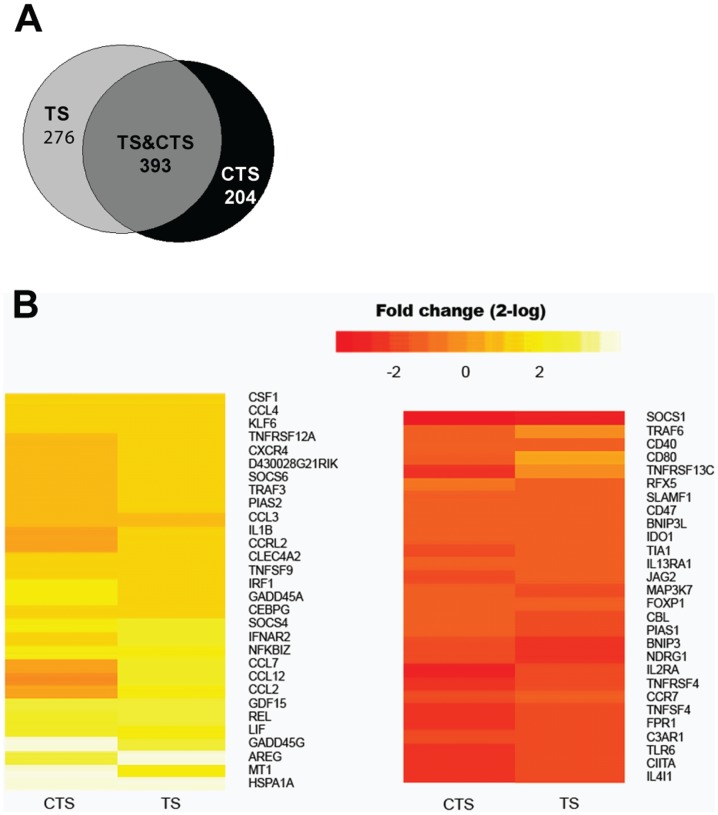
Gene expression profile of TS and carvacrol-TS treated BMDC as compared to untreated BMDC. Mouse BMDC were incubated with 0.1 mM carvacrol or were left untreated. After two hours a one hour TS at 42.5°C followed. Samples for microarray analysis were taken 0.5 hour after TS. **A.** Venn diagram showing genes differentially expressed (p≤0.05) with at least a two-fold change for TS treated BMDC (left circle) and carvacrol-TS treated BMDC (right circle) compared to untreated BMDC. The numbers in the circles indicate the modulated genes for each condition. The grey area represents the overlap between both conditions. **B.** Heat map of immunological relevant genes that were significantly (p≤0.05) differentially expressed with at least a two-fold change in carvacrol-TS and/or TS treated BMDC compared to untreated BMDC. The heat map shows the log-ratio of the mean of three independent experiments.

Previously we have shown that carvacrol in a concentration of 0.1 mM was a strong co-inducer of HSP70 in various antigen presenting cells [8], [18]. To study whether carvacrol pretreatment besides affecting the heat shock response, also altered the immunological phenotype of DCs we performed a microarray analysis on BMDC. Similar to the HSP70 co-inducing effect, carvacrol treatment alone, without additional stress did not induce any differentially expressed genes compared to untreated BMDC (data not shown). Conversely, BMDC treated with carvacrol in combination with TS or TS alone led to differential expression of approximately 870 genes with at least a two-fold difference when measured 0.5 hour following TS (figure 1a). Since, the differential expression of many of these genes most likely reflected the impact of the TS treatment we focused on the immunologically relevant genes as indicated by DAVID analysis (figure 1b). Of the fifty-eight immunologically relevant genes, genes with a high fold change or genes with particular interest for DCs were selected for further research (table 2).

**Table 2:** Genes with a high fold change or particularly interesting for DCs.

*Chemokines*		F.C. TS	F.C. CTS
CCL2	chemokine (C-C motif) ligand 2	2.97	1.09
CCL7	chemokine (C-C motif) ligand 7	5.24	0.99
CCL12	chemokine (C-C motif) ligand 12	4.91	1.18
*Cytokines*			
IL-1β	interleukin 1 beta	2.36	1.44
LIF	leukemia inhibitory factor	3.77	5.02
AREG	amphiregulin	13.25	5.67
*Stress induced immunologically relevant molecules*			
MT1	metallothionein 1	3837.54	3795.52
HSP1A1	heat shock protein 1A	3.65	50.28
*Maturation markers*			
IL-2Rα (CD25)	interleukin 2 receptor, alpha chain	0.33	0.21
CD40	CD40 antigen	0.59	0.47
CD80	CD80 antigen	0.68	0.48

BMDC were treated as in figure 1. Genes that are particularly interesting for DC were selected and fold changes (F.C.) of gene expression in TS BMDC treated with or without carvacrol compared to that of fully untreated BMDC are shown.

### DC fingerprint after TS and carvacrol-TS treatment

#### Increased expression of CCR2 binding chemokines

**Figure 2:**
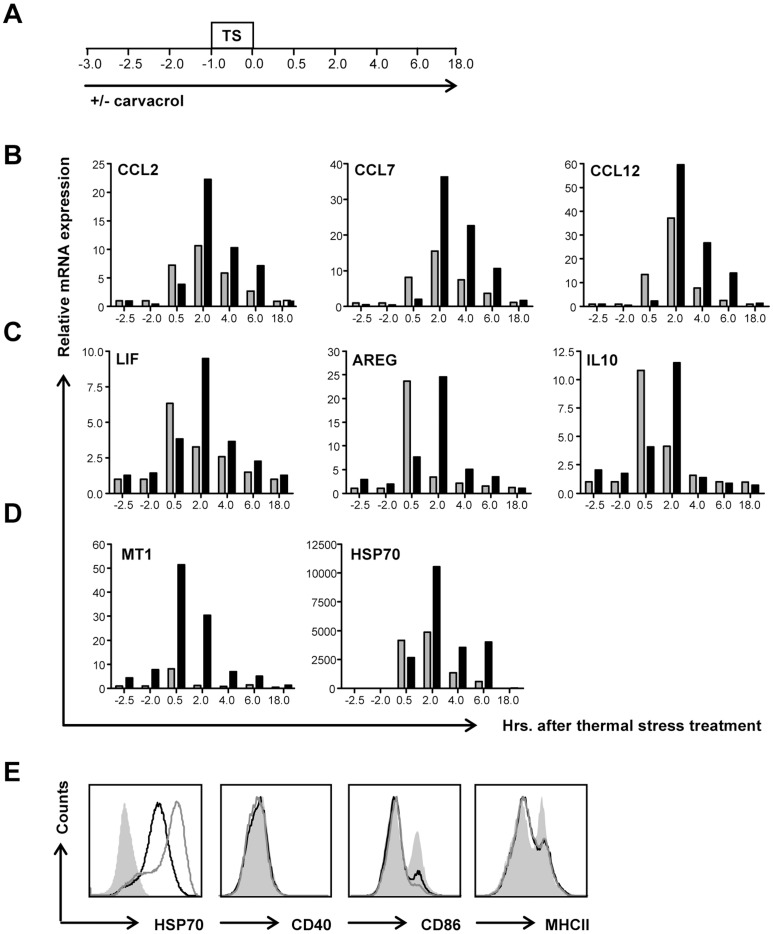
DC fingerprint after TS and carvacrol-TS treatment. Mouse BMDC were incubated with 0.1 mM carvacrol or were left untreated. After two hours a one hour TS at 42.5°C followed. **A**. Experimental setup. **B-D:** Cells were collected at indicated time points and mRNA was isolated. With cDNA quantitative RT-PCRs were performed in untreated, TS treated (grey) and carvacrol-TS treated (black) BMDC. Results were depicted relative to untreated BMDC at the indicated time points. Data are representative of three independent experiments. **B.** Quantitative RT-PCRs for CCL2, CCL7 and CCL12. **C.** Quantitative RT-PCRs for LIF, AREG and IL-10. **D.** Quantitative RT-PCRs for HSP70 and MT1. **E.** After ON recovery at 37°C, intracellular HSP70 levels and extracellular CD40, CD86 and MHCII were analyzed. Grey solid: untreated BMDC; black line: TS treated BMDC; grey line: carvacrol-TS treated BMDC. Results are representative of at least 5 independent experiments.

Chemokines CCL2, CCL7 and CCL12 were upregulated after TS treatment but not with carvacrol-TS treatment measured 0.5 hour after TS (table 2). These β-chemokines all bind to the chemokine receptor CCR2 which is expressed on several different immune cells like monocytes, T cell subsets and granulocytes. To study expression profiles of CCL2, CCL7 and CCL12 in more detail mRNA levels were measured by quantitative RT-PCR in a kinetics experiment. As shown in figure 2b, mRNA levels in TS treated BMDC were upregulated after TS. Interestingly, in carvacrol-TS treated BMDC upregulation of chemokine mRNA was delayed, but eventually much higher expression levels were reached. As a control, thymol and p-cymene, two compounds structurally similar to carvacrol (figure S1a), were also tested for the induction of CCL2. As shown in figure S1c p-cymene-TS treated BMDC did not alter CCL2 mRNA expression as compared to TS treated BMDC. In contrast, thymol-TS treatment induced CCL2 expression comparable to carvacrol-TS treatment. However, the duration of the elevated expression levels was shorter.

#### Differentially expressed gene expression of RA relevant cytokines

Important mediators in the pathogenesis of RA are the pro-inflammatory cytokines IL-6, IL-1β and TNFα. Additionally, other less well known cytokines like leukemia inhibitory factor (LIF) and amphiregulin may also contribute to joint inflammation [25], [26]. The mRNA expression levels of IL-1β, LIF and amphiregulin were upregulated in TS as well as carvacrol-TS treated BMDC, with a delay but eventually higher expression levels in BMDC pretreated with carvacrol (table 2, figure 2c and data not shown). mRNA expression levels of the anti-inflammatory cytokine IL-10 were also upregulated after both treatments and followed the same expression profile as IL-1β, amphiregulin and LIF (figure 2c). In conclusion, mRNA expression levels of pro-inflammatory RA relevant cytokines as well as the anti-inflammatory cytokine IL-10 were increased after treatment. In the controls where BMDC were treated with thymol-TS or p-cymene-TS, amphiregulin mRNA expression was not altered as compared to TS. IL-10 expression levels were only increased after thymol-TS treatment as compared to TS treatment (figure S1c).

#### Induction of immunoregulatory stress related molecules

TS induces the expression of many stress related genes and some of these, like metallothionein 1 (MT1) and HSP70 [27] are also considered to be immunomodulatory molecules [28], [29]. As shown in table 2 and figure 2d, expression levels of MT1 were upregulated after TS treatment but especially after carvacrol-TS treatment. Interestingly although not detectable in the microarray, in non-stressed carvacrol treated BMDC mRNA levels of MT1 were also increased (figure 2d). Expression levels of HSP70 mRNA and protein increased shortly after both TS and carvacrol-TS treatment. However, carvacrol enhanced and prolonged expression levels (figure 2d and e). In sum, expression of the immunoregulatory stress related genes MT1 and HSP70 was elevated in TS and especially in carvacrol-TS treated BMDC, suggesting the induction of DCs with a relatively tolerogenic phenotype. The structurally similar compound thymol was also able to act as a co-inducer and elevated expression levels of HSP70 and MT1. Nevertheless, expression levels were lower and duration of expression was shorter as compared to carvacrol-TS treatment. Pretreatment of DCs with p-cymene did not alter mRNA expression levels as compared to TS alone (figure S1c and d).

#### Reduced maturation

As maturation status is an important hallmark for tolerogenic DC and the activation markers CD40, CD80 and CD25 (IL2rα) [30] were downregulated in the microarray (table 2), we assessed protein expression of activation markers on treated and untreated BMDC. TS treated BMDC displayed an immature phenotype with lower expression levels of CD86 and CD25 and MHC class II as compared to untreated BMDC. In addition, carvacrol-TS treatment could even further decrease expression of CD86 and CD25 in BMDC. In contrast, protein expression of CD40 and CD80 (figure 2e and data not shown) were not altered. No differences could be detected either in annexin V/7AAD staining or Dextran-FITC uptake respectively (data not shown), showing that reduced maturation is not the result of reduced viability or function of the cells.

### Suppressed proliferation and activation of antigen specific T cells

**Figure 3:**
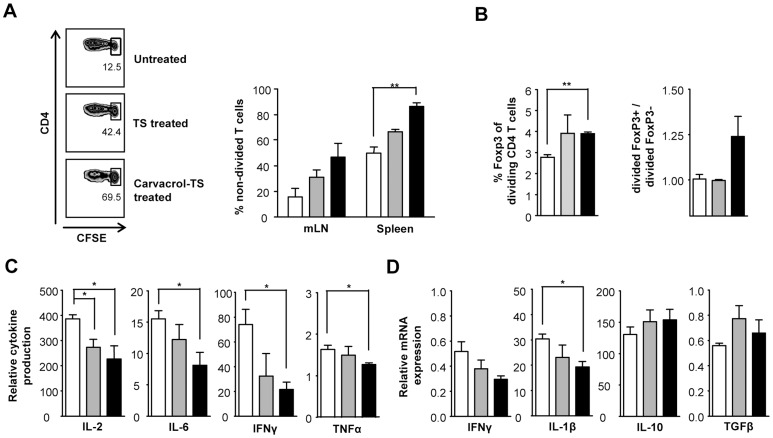
Carvacrol-TS treated BMDC induce a less pro-inflammatory T cell. Mice were intravenously injected with 1×10^7^ CFSE labeled pOVA-specific T cells one day prior to intraperitoneally injection of 1×10^6^ pOVA pulsed untreated, TS treated or carvacrol-TS treated BMDC. Four days after BMDC injection spleen and mLN were harvested. **A.** Representative examples of CFSE dilution of pOVA-specific T cells in mLN (left) and percentage of undivided pOVA-specific T cells in spleen and mLN (right). **B.** Percentage of FoxP3 expression of pOVA-specific proliferating T cells (left) and percentage of replicated FoxP3^+^ cells of total FoxP3^+^ cells divided by the percentage of replicated FoxP3^−^ cells of total FoxP3^−^ cell population (right). Data are analyzed by flow cytometry. **C-D.** Splenic cell suspensions were restimulated with pOVA for 72 hours. Cytokine secretion for IL-2, IL-6, IFNγ and TNFα relative to non-stimulated cells are shown (C). Cytokine expression of IFNγ, IL-1β, IL-10 and TGFβ was measured by quantitative RT-PCR. Data are expressed as relative expression to the calibrator HPRT (D). White: untreated BMDC, grey: TS treated BMDC, black: carvacrol-TS treated BMDC. Values are the mean and SEM of three mice per group. * (p<0.05) and ** (p<0.01).

TS and carvacrol-TS treated BMDC have increased mRNA expression levels of certain anti-inflammatory mediators and display a decreased maturation profile. To investigate whether the assorted phenotype we observed in mRNA expression of the BMDC resulted in an immunomodulatory function of treated BMDC we studied their antigen presenting capacity *in vivo*. pOVA-specific CFSE-labeled CD4^+^ T cells were intravenously transferred into recipient mice one day prior to intraperitoneal injection of treated and untreated BMDC pulsed with pOVA. Three days later proliferation and phenotype of the pOVA-specific T cells from spleen and mLN were analyzed. Transfer of TS treated BMDC induced less T cell proliferation as compared to untreated BMDC *in vivo*. Notably, carvacrol-TS treated BMDC induced even lower proliferation *in vivo* as shown by the increase in non-divided cells (figure 3a). In addition, although carvacrol-TS treated BMDC did not increase FoxP3 expression in the total CD4^+^DO11.10^+^ population, mice treated with carvacrol-TS treated BMDC had increased FoxP3 expression levels in the proliferating CD4^+^DO11.10^+^ population in the mLN as compared to untreated BMDC (figure 3b). Moreover, carvacrol-TS treated BMDC preferentially induced the proliferation of FoxP3^+^ T cells over proliferation of FoxP3^−^ T cells. In contrast, with untreated and TS treated BMDC the percentage of replicated FoxP3^+^ T cells of the total FoxP3^+^ T cells was equal to the replicated FoxP3^−^ T cells of the total FoxP3^−^T cells (figure 3b). Results did not reflect a difference in survival of the treated BMDC as equal amounts of transferred viable BMDC could be recovered in spleens of recipient mice that received untreated, TS treated or carvacrol-TS treated BMDC (data not shown). To further investigate the phenotype of the DC primed T cells, splenic cell suspensions were *ex vivo* restimulated with pOVA. Secretion of the pro-inflammatory cytokines IL-2, IL-6, IFNγ and TNFα was reduced when mice were injected with TS or carvacrol-TS treated BMDC as compared to untreated BMDC. However, reduction was only significantly lower with carvacrol-TS treated BMDC (figure 3c). Furthermore, mRNA expression levels of the pro-inflammatory cytokines IFNγ and IL-1β were downregulated, whereas expression levels of IL-10 and TGFβ were slightly upregulated (figure 3d). Thus, these data indicated that treated BMDC induced less activated and less pro-inflammatory T cells and that carvacrol-TS treated BMDC as compared to untreated BMDC were able to enhance the proportion of proliferating FoxP3^+^ T cells.

### Pretreatment of BMDC with carvacrol is essential for suppression of arthritis

**Figure 4:**
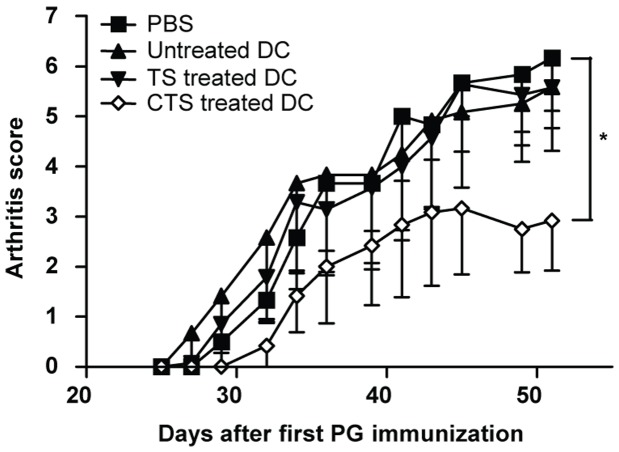
Carvacrol-TS treated BMDC suppress PGIA. Arthritis was induced by two intraperitoneally immunizations with human PG on days 0 and 21. One day prior to the second PG immunization mice were intraperitoneally injected with 1×10^6^ PG pulsed untreated, TS treated or carvacrol-TS treated BMDC. As a control, mice received only PBS. Arthritis severity expressed as the mean and SEM of at least six mice per group. * (p<0.05) for area under the curve compared to PBS group.

Both TS and carvacrol-TS treatment protocols induce a BMDC with an assorted gene expression profile that is able to suppress proliferation and activation of antigen-specific T cells. To determine whether these treated BMDC can suppress PGIA, mice were intraperitoneally injected with carvacrol-TS treated BMDC, TS treated BMDC, untreated BMDC or PBS one day prior to the second PG immunization. PG pulsed and unpulsed BMDC were compared to distinguish if loading of DCs with an auto-antigen was required. As shown in figure 4 and table 3, only carvacrol-TS treated BMDC that were pulsed with PG significantly reduced arthritis scores and delayed disease onset in recipient mice. In summary, although TS treated BMDC show characteristics of a tolerogenic DC, they are not anti-inflammatory in PGIA, whereas carvacrol-TS treated BMDC pulsed with disease associated antigens were able to suppress autoimmune arthritis.

**Table 3:** Mean day of onset and arthritis score.

	PG pulsed	Day of onset	Maximum arthritis score
**PBS**		30±1.3	8.3±1.25
**Untreated BMDC**	+	36±2.8	6.9±1.16
**TS treated BMDC**	+	33±1.9	6.9±1.39
**Carvacrol TS treated BMDC**	+	38±3.1*	3.8±1.26*
**Untreated BMDC**	−	29±1.0	8.3±1.16
**TS treated BMDC**	−	30±2.0	5.5±1.48
**Carvacrol TS treated BMDC**	−	34±2.9	5.5±1.37

To test the tolerogenic capacity of carvacrol-TS treated BMDC mice were treated as in figure 4, although here, PG-unpulsed BMDC recipients are also shown. Values indicate mean ± SEM of n = 6 (PBS, untreated BMDC-pulsed, carvacrol-TS treated BMDC-pulsed and untreated BMDC-unpulsed) or n = 7 (TS treated BMDC-pulsed, TS treated BMDC-unpulsed and carvacrol-TS treated BMDC-unpulsed). * (p≤0.05) compared to PBS group.

## Discussion

Approaches to induce tolerogenic phenotypes in DCs have been developed and used successfully in autoimmune models [1], [31], [32]. Earlier we found that oral administration of carvacrol suppressed PGIA in a T cell dependent manner [8]. Furthermore, carvacrol had an anti-inflammatory effect on activated macrophages [15]. In this study, we demonstrated that treatment with carvacrol and TS induced a functional tolerogenic DC that, upon transfer, was able to suppress autoimmune arthritis.

The immunological effect of carvacrol on DCs was analyzed by microarrays. We found that carvacrol acted as co-inducer as no effects in the absence of TS were seen 0.5 hour after carvacrol administration. We used severe TS as stimulus as it is known that carvacrol functions as co-inducer for the heat shock response [8], [18]. Controversy exists as both immune activating and inhibitory effects have been described for mild TS [10], [12], however the effects of severe TS comparable to our TS protocol on the immune response are less well known. Fifty-eight immunologically relevant genes were differentially expressed with at least a two-fold change and a p-value ≤0.05, 0.5 hour after TS and/or carvacrol-TS treatment compared to untreated BMDC (figure 1b). Although most of these genes are altered after both treatment protocols, differences were found in the timing of expression and expression levels as shown by table 1 and figure 2. Especially the lower expression levels of certain maturation markers and the excessively high levels of potentially regulatory stress molecules could steer towards a tolerogenic functional phenotype in carvacrol-TS treated BMDC as compared to TS treatment alone.

One group of genes with elevated expression levels in treated BMDC were the CCR2 binding chemokines CCL2, CCL7 and CCL12. These chemokines can have diverse effects on the immune response as they attract both pro-inflammatory cells and also CCR2^+^FoxP3^+^CD4^+^ regulatory T cells. CCR2^+^ regulatory T cells are important regulators in the progression phase of collagen induced arthritis (CIA) [33]. Furthermore, CCL2 and CCL7 promote a Th2 response as CCR2^+^ DCs produce less IL-12 and activated CCR2^+^ T cells secrete more IL-4 after binding to CCL2. [34], [35]. Enhanced expression of these chemokines might promote Th2 or regulatory T cell pathways and thereby suppress arthritis, depending on the micro-environmental context in which the chemokines are released.

A second group of genes that showed differential expression were certain RA relevant cytokines. The IL-1 family member IL-1β and the IL-6 family member LIF were two genes with elevated expression levels after both TS and carvacrol-TS treatment in BMDC. These pro-inflammatory cytokines contribute to joint inflammation in RA and are not directly indicative of a tolerogenic phenotype. In contrast, the expression levels of anti-inflammatory cytokine IL-10 and amphiregulin were increased. Amphiregulin is increased in the synovial tissue of RA patients [26], but is also described as an immunomodulatory molecule that is secreted by ATP treated semi-mature tolerogenic DCs. [36].

Expression levels of the immunomodulatory stress related molecules HSP70 and MT1 were elevated after both treatments, although up-regulation was more prominent after carvacrol-TS treatment. This result is in concordance with the co-inducing capacity of carvacrol on the stress response. HSP70 functions as an intracellular chaperone, but has also been described as an anti-inflammatory molecule that inhibits maturation and induces the secretion of IL-10 in DCs [28], [37]. MT1 is a protein that binds heavy metals and functions as an immunosuppressive agent on macrophages and T and B cells [38]–[40]. Moreover, repeated administration of MT1 and 2 during the course of CIA dramatically reduced incidence and disease severity via induction of TGFβ and down-regulation of pro-inflammatory mediators [29] and it was shown to induce IL-10 and TGFβ producing regulatory T cells in CIA [41]. Elevated expression levels of both molecules point toward a tolerogenic DC that functions via the induction of anti-inflammatory cytokines like IL-10 and TGFβ.

The capacity of carvacrol to enhance the stress response and the expression of certain immunoregulatory mediators induced by TS is not a unique feature of carvacrol as the structurally similar compound thymol could also affect some of these same genes. However, carvacrol was the most potent co-inducer of the immune response and had the largest effect on the expression of immunological relevant genes making carvacrol the most suitable compound.

It is clear that our microarray analysis of TS and carvacrol-TS treated BMDC revealed a diverse gene expression profile with potential tolerogenic features. In addition, the diverse nature and kinetics of the 58 immunologically relevant genes could at least partly help explain why there exists such a controversy in literature about thermal stress as a pro- or anti-inflammatory trigger.

Subsequently, maturation status of treated BMDC was investigated as a possible feature of tolerogenic DCs. Maturation was decreased after TS treatment and even stronger after pretreatment with carvacrol. Both TS and carvacrol-TS treated BMDC induced proliferation of CD4^+^ T cells, although less compared to untreated BMDC. This reduced proliferation coincided with reduced secretion of pro-inflammatory cytokines following 72 hours of restimulation indicating that the antigen specific T cells had changed *in vivo* due to BMDC treatment. In addition, differences were more pronounced in carvacrol-TS treated BMDC and only carvacrol-TS treated BMDC preferentially induced the proliferation of FoxP3^+^ T cells over the proliferation of FoxP3^−^ T cells. These data suggested proliferation of existing FoxP3^+^ natural regulatory T cells or the induction of a regulatory phenotype in naïve T cells. These combined results are an indication that an anergic or regulatory T cell response was induced.

In conclusion, this study shows that although TS and carvacrol-TS treated BMDC share several anti-inflammatory characteristics, only carvacrol pretreated BMDC can function as tolerogenic DC in suppressing arthritis. This is probably caused by differences in the balance between pro- and anti-inflammatory molecules. For example, both TS and carvacrol-TS treated BMDC express the pro-inflammatory cytokines LIF and IL-1β, two mediators involved in the pathogenesis of RA. In contrast, carvacrol-TS treated BMDC also express very high levels of the immunoregulatory molecules MT1 and HSP70, whereas TS treated BMDC express lower levels of both molecules. These differences probably cause the shift toward a tolerogenic function after carvacrol-TS treatment, but not after TS treatment alone. Our data show that the combined action of a phytonutrient carvacrol, in combination with a physiological form of stress, can lead to the induction of a tolerogenic state in DCs.

## Supporting Information

Figure S1 **Mouse BMDC were incubated with 0.1 mM carvacrol, 0.1 mM thymol, 0.1 mM p-cymene or were left untreated.** After two hours a one hour TS at 42.5°C followed. **A**. Chemical structures of cavacrol, thymol and p-cymene. **B.** Experimental setup. **C.** Cells were collected at indicated time points and mRNA was isolated. With cDNA quantitative RT-PCRs were performed in untreated, TS treated (grey), p-cymene-TS treated (white), thymol-TS treated (white striped) and carvacrol-TS treated (black) BMDC. Results were depicted relative to untreated BMDC at the indicated time points. **D.** After over night recovery at 37°C, intracellular HSP70 levels were analyzed. Grey solid: untreated BMDC; black line: TS treated BMDC; grey line: p-cymene/thymol/carvacrol-TS treated BMDC.
(TIF)
