# Supplementary figures and images for: Tolerogenic Dendritic Cells That Inhibit Autoimmune Arthritis Can Be Induced by a Combination of Carvacrol and Thermal Stress
Source: PLoS One. 2012 Sep 25;7(9):e46336. doi: 10.1371/journal.pone.0046336 (PMC3457998; doi:10.1371/journal.pone.0046336)

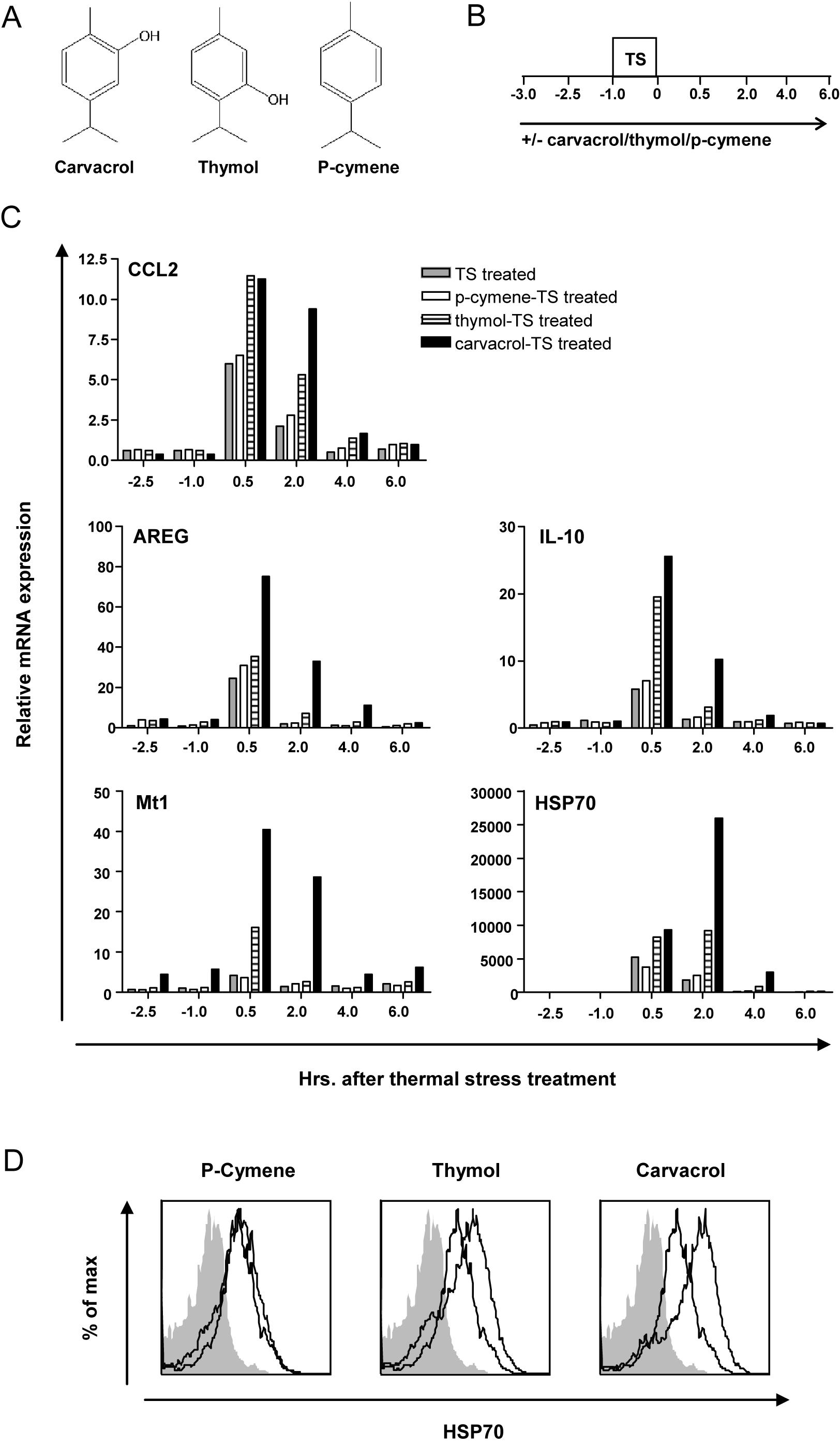

Supplement: Figure S1 — Mouse BMDC were incubated with 0.1 mM carvacrol, 0.1 mM thymol, 0.1 mM p-cymene or were left untreated. After two hours a one hour TS at 42.5°C followed. A. Chemical structures of cavacrol, thymol and p-cymene. B. Experimental setup. C. Cells were collected at indicated time points and mRNA was isolated. With cDNA quantitative RT-PCRs were performed in untreated, TS treated (grey), p-cymene-TS treated (white), thymol-TS treated (white striped) and carvacrol-TS treated (black) BMDC. Results were depicted relative to untreated BMDC at the indicated time points. D. After over night recovery at 37°C, intracellular HSP70 levels were analyzed. Grey solid: untreated BMDC; black line: TS treated BMDC; grey line: p-cymene/thymol/carvacrol-TS treated BMDC. (TIF) [file pone.0046336.s001.tif]
